# Overview of Methylation and Demethylation Mechanisms and Influencing Factors of Mercury in Water

**DOI:** 10.3390/toxics12100715

**Published:** 2024-09-30

**Authors:** Wenyu Zhao, Runjie Gan, Bensen Xian, Tong Wu, Guoping Wu, Shixin Huang, Ronghua Wang, Zixuan Liu, Qin Zhang, Shaoyuan Bai, Mingming Fu, Yanan Zhang

**Affiliations:** 1 College of Environmental Science and Engineering, Guilin University of Technology, Guilin 541004, China; zhaowenyu@glut.edu.cn (W.Z.); xianbensen@glut.edu.cn (B.X.); szxbclgysmyd@163.com (T.W.); liuzixuaniiiii@163.com (Z.L.); qinzhanggl@163.com (Q.Z.); baisy@glut.edu.cn (S.B.); mmfu@glut.edu.cn (M.F.); 2 Collaborative Innovation Center for Water Pollution Control and Water Safety in Karst Area, Guilin University of Technology, Guilin 541004, China; 3 Guangxi Beitou Environmental Protection & Water Group Co., Ltd., Nanning 530025, China; 4 Ecological Environment Monitoring Station of Shunde, Foshan 528399, China; sdwugp@163.com (G.W.); huangshixin623@163.com (S.H.); 5 Hengsheng Water Environment Treatment Co., Ltd., Guilin 541100, China

**Keywords:** mercury, methylmercury, methylation, demethylation, water

## Abstract

Mercury, particularly in its methylated form, poses a significant environmental and health risk in aquatic ecosystems. While the toxicity and bioaccumulation of mercury are well documented, there remains a critical gap in our understanding of the mechanisms governing mercury methylation and demethylation in aquatic environments. This review systematically examines the complex interplay of chemical, biological, and physical factors that influence mercury speciation and transformation in natural water systems. We provide a comprehensive analysis of methylation and demethylation processes, specifically focusing on the dominant role of methanogenic bacteria. Our study highlights the crucial function of hgcAB genes in facilitating mercury methylation by anaerobic microorganisms, an area that represents a frontier in current research. By synthesizing the existing knowledge and identifying key research priorities, this review offers novel insights into the intricate dynamics of mercury cycling in aquatic ecosystems. Our findings provide a theoretical framework to inform future studies and guide pollution management strategies for mercury and its compounds in aquatic environments.

## 1. Introduction

Mercury is a worldwide both natural and man-made pollutant that can be widely dispersed in nature through a complex conversion and transport process. In most environments, mercury is present as elemental mercury, inorganic divalent mercury, and organic Hg compounds (e.g., MeHg). Each form of mercury poses a different health hazard, depending on the dose and route of exposure. Methylmercury is currently one of the most concerning compounds worldwide because this organic mercury compound is highly bioaccumulative and has been shown to have neurotoxic effects on humans, especially in the early stages of brain development [1,2]. In addition, exposure to methylmercury can also be transmitted from mother to newborn, and even in developed countries like the United States, tens to hundreds of thousands of children are born each year with intrauterine methylmercury exposures above the prescribed limit of health guidelines. Maternal fish consumption is thought to be the primary route of methylmercury exposure in newborns [3].

Methylmercury is the most toxic form of mercury. Deeply studying the pathways of formation and decomposition of methylmercury is crucial for predicting environmental levels of methylmercury and its bioavailability. Methylation and demethylation processes in aquatic environments significantly influence methylmercury levels. Key microorganisms, including sulphate-reducing, iron-reducing, and methanogenic bacteria, drive mercury methylation. Chemomethylation may also occur with available methyl donors. Demethylation can occur through biotic agents, such as mercury-resistant bacteria with mer genes, and abiotic pathways, like photodemethylation, which is most effective in surface water due to UV radiation. This article reviews the mechanism of methylation and demethylation of mercury in aquatic environments and summarizes the influencing factors of mercury methylation and demethylation in aquatic environments.

## 2. Methylation of Mercury in the Aquatic Environment

Mercury in the aquatic environment is mainly methylated in the sediments of the water system, and sediment mercury methylation is the main source of methylmercury [4,5]. This effect is also present in water and epiphytes, as there are studies showing that the methylation of mercury can occur in seawater, freshwater, and epiphytes [6,7,8,9]. In addition, inorganic Hg can be methylated to methylmercury in the water environment by either the biotic pathway [7,8,9,10,11,12,13,14] or the abiotic pathway (photo- or non-photo-mediated chemical methylation) [15,16,17,18,19,20,21,22,23,24,25,26,27,28,29]. The biological pathway is generally considered to be the major route for the methylation of inorganic Hg in natural aquatic environments.

Excessive methylation in water bodies is, in turn, a potential cause of many environmental problems. As early as the 1950s, there were reports of sporadic outbreaks of encephalopathy of unknown cause in Minamata Bay, Japan. Numerous studies have demonstrated that this disease is caused by prolonged methylmercury exposure in humans or by its ingestion in large quantities, and has been named Minamata disease [30]. The reason for this is that the wastewater discharged from the factories near Minamata Bay contains large amounts of mercury, which is converted to methylmercury in aquatic ecosystems by aquatic organisms and finally ingested by humans, resulting in methylmercury poisoning [31]. Methylmercury is highly toxic, and although it is being controlled worldwide, it is still present in many common substances and may threaten human health, as shown in Table 1.

### 2.1. Microbial Pathway Methylation

**Figure 1:**
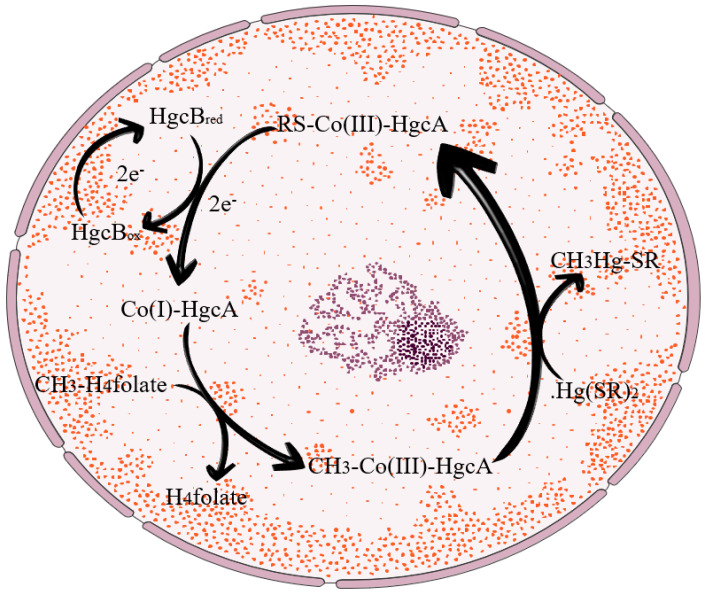
Mechanism of action of HgcAB gene pairs within anaerobic bacteria.

By the 1970s, different studies had identified biomethylation of inorganic Hg in the sediments of Swedish rivers, lakes, and coastal waters [40]. Afterwards, many researchers conducted studies on the above findings. Methylation of mercury by biological pathways might occur either enzymatically or non-enzymatically. Enzymatic methylation of mercury requires the existence of active metabolizing organisms, whereas non-enzymatic methylation of mercury requires only the products of active metabolic methylation. The detailed mechanism of mercury methylation was first investigated and discovered by Wood et al. [41]. This study found that methylcobalamin, a derivative of vitamin B_12_ generated in numerous living organisms, is involved in the process of microbial mercury methylation. It was also proposed that this procedure is a non-enzymatic shift of methylcobalamin to mercury ions. De Simone et al. [42] demonstrated that the shift of methyl towards Hg^2+^ is a methylation (CH_3_^−^) process. Although many molecules are potential methyl donors in the aquatic environment, methylcobalamin is the only naturally occurring methylation reagent that can transfer methyl in the form of carbon ions, and is representative of the non-enzymatic biological methylation of mercury [43]. This ion transfer process is prevalent in anaerobic ecosystems and organisms. Methylcobalamin produced by microbial metabolism can cause spontaneous methylation of Hg^2+^ in aqueous solutions [44] and is a major supplier of methylmercury in water. In addition, microorganisms capable of methylating Hg have also all been found in anaerobic, facultative anaerobic, and aerobic bacteria. However, the potential for mercury methylation by microorganisms is often greater during periods of anaerobic conditions. It has been demonstrated that the hgcA and hgcB genes regulate the process of mercury methylation in microorganisms and are the signature genes of Hg-methylated microorganisms. Parks et al. demonstrated the critical importance of the hgcA and hgcB genes in the mercury methylation process in some anaerobic bacteria, with the deletion of the hgcA and hgcB genes leading to changes in the physiological properties of the cell surface and affecting mercury interactions [45]. These genes encode a putative protease, hgcA, and a 2[4Fe-4S] ferredoxin, hgcB, with roles as methyl carrier and electron donor, respectively, required for protease cofactor reduction. The specific mechanism of action of the hgcA and hgcB genes is shown in Figure 1. In this study, the hgcA and hgcB gene clusters in two bacteria, Desulfovibrio desulfuricans ND132 and Geobacter sulfurreducens PCA, were required genes for mercury methylation. In both bacteria, the deletion of hgcA or hgcB resulted in the loss of mercury methylation. Further studies by Hui Lin and Richard A. Hurt, Jr et al. found that deleting hgcA and hgcB genes increased the rate of Hg(II) reduction and decreased the oxidation ratio of Hg(0), leading to the loss of methylation activity [46]. In additional studies involving a 40-kDa corrinoid protein in enzymatic Hg methylation, enzymatic methylation was more favoured when pH = 4.0 and the temperature was 32 degrees Celsius [47,48].

Sulphate-reducing bacteria have been identified as the major methylating factor for inorganic Hg in anaerobic sediments [49,50,51]. The methylation of Hg^2+^ by sulphate-reducing bacteria (SRB) in aquatic sediments was confirmed for the first time by Compeau and Bartha [52]. In this study, their findings showed that in the presence of sodium molybdate, which is a site-specific suppressant of sulphate reductase, the methylation of Hg^2+^ in hypoxic sediments was reduced by more than 95%. Choi and Bartha et al. [53] confirmed the role of methylcobalamin as a methyl-donating agent when desulfovibrio desulfuricans (LS strains) methylates Hg^2+^. In addition, it is possible that the mercury methylation is dominated by other microorganisms, even if this may occur only in a restricted number of aquatic ecosystems. For example, ferri-reducing bacteria with the ability to methylate mercury have been identified in both natural sediments and pure cultures [54].

Recent investigations suggest a dominant effect of methanogens in the methylation of mercury in river, lacustrine, and marine deposits. Yuwei Wang and Spencer Roth et al. studied estuarine sediments collected from the San Jacinto River and found that methylmercury concentrations were reduced by a factor of 12 when the sediment was treated with the addition of the methanogenic inhibitor 2-bromoethanesulphonate (BES). The dominant effect of methane-producing bacteria in the methylation of mercury has been confirmed in polluted rivers [55]. Geoff A. Christensen et al., in their analyses of stream sediments from eastern Oak Ridge, Tennessee, found that the abundance of methanogenic bacteria was 2–5 times higher than that of other Hg-methylating microorganisms, suggesting that methanogenic bacteria may be the main Hg-methylating microorganisms in the river sediment [56].

### 2.2. Abiotic Pathway Methylation

Pure chemomethylation of mercury is also possible if a proper methyl donor is available. Celo et al. demonstrated that the reaction of water-soluble methyl–silica complexes with Hg^2+^ resulted in the formation of methylmercury [57]. In addition, organosiloxane and other silicon-related substances have also been considered as possible methylation reagents [58]. Under photochemically induced conditions, mercuric chloride can undergo alkylation reactions from methanol, ethanol, acetic acid, and propionic acid [29]. Moreover, domestic sewage and industrial effluents might also be the source of methyl for the photochemical methylation of mercury.

Mercury methylation may also be the result of transmethylation reactions between mercury and alkyltin and lead used as gasoline additives, with trimethyllead chloride and trimethyltin chloride both transferring methyl to Hg^2+^ [59]. Trimethyllead was identified as an especially potent methylator of mercury, and higher methylmercury concentrations in sediments may be caused by transmethylation reactions induced by alkyl lead releases. Humic substances may also be another important environmental methylator, and the ability of abiotic factors such as humic compounds to form methylmercury has been demonstrated in different studies [47]. In addition, amino acids and low molecular organic acids that can methylate inorganic mercury under laboratory conditions have been found, and some of these reactions require radiation as an experimental condition [60,61]. However, the occurrence of these reactions in the natural environment is likely to be low, and their importance has not been demonstrated in the natural environment. Additionally, the main current difficulty may be that laboratory conditions cannot fully simulate realistic natural environments. Because many variables are constantly changing in the natural environment, it can be very difficult to control these variables in the laboratory to simulate the real environment. While the comparative significance of biotic versus abiotic processes of methylation within the natural aquatic setting has not yet been determined, it is commonly accepted that Hg methylation is primarily a microbial process.

## 3. Demethylation of Mercury in Aquatic Environments

**Figure 2:**
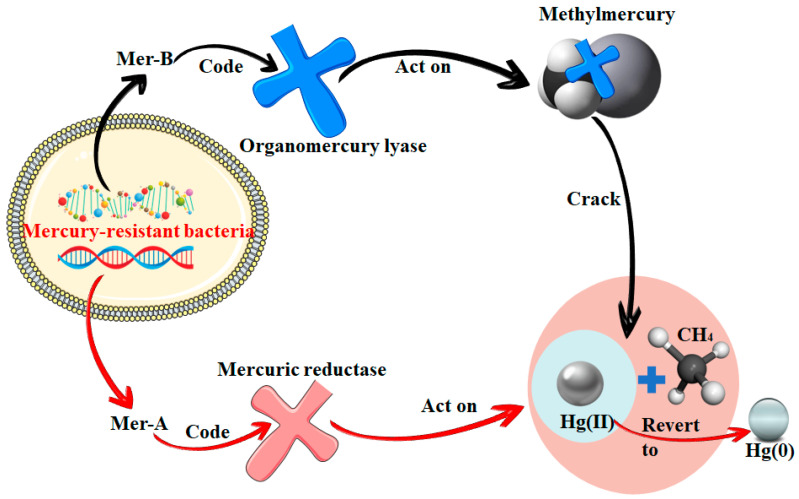
Mer-B and Mer-A genes dominate the demethylation process.

The demethylation of methylmercury can also occur via biotic or abiotic pathways (photodemethylation or non-photodemethylation). The difference between photodemethylation and non-photodemethylation is mainly whether or not light is involved. Methylmercury can be photodegraded either directly or indirectly by photolytic or photochemical reactions, a mechanism known as photolytic methylation, which currently appears to be the most important abiotic decomposition mechanism. Biological functions are the main pathway for mercury demethylation in sediments and epiphytes. It has been shown that heavily polluted sediments usually contain populations of microorganisms that positively degrade methylmercury via mer-denitrification and that oxidative demethylation also occurs in heavily polluted sediments, whereas oxidative demethylation plays a dominant role in less polluted sediments [62]. Marvin-DiPasquale M and Agee J et al. found that mercury-resistant bacteria possessing genes for mer manipulators can dominate the demethylation process and that this ability is widespread in nature [63]. In broad-spectrum mercury-tolerant bacteria, the mer-B gene can encode an organomercury lyase that cleaves methylmercury to form CH_4_ and Hg(II), and the mer-A gene would encode a mercuric reductase that further reduces Hg(II) to the volatile element Hg(0) [18]. However, this methylmercury degradation route is only available in some aerobic prokaryotes. The specific mechanism of action of the Mer-A and Mer-B genes is shown in Figure 2.

Sulphate-reducing bacteria (SRB) and methanogens may be the main microorganisms involved in anaerobic demethylation, and the products of anaerobic demethylation are mainly CO_2_ and, to a lesser extent, CH_4_ [64,65,66]. Xia Lu and Wenyu Gu et al. found in their experiments the existence of a new pathway for the biodegradation of methylmercury by methanogens that is distinctly different from typical organic Hg lyases found in some aerobic microorganisms, and that the methyl group in methylmercury can be used as an auxiliary C1 source for methanogens [67]. Although microbial demethylation of methylmercury has been observed in the water column or in epiphytes and phytoplankton may degrade methylmercury in the presence of solar radiation [68], in phytoplankton cells, approximately as much as 36–85% of the methylmercury may undergo degradation to either inorganic Hg(II) and/or Hg(0) via dark reactions [69]. However, the most important demethylation function in the water column is photodemethylation [70,71]. Non-photo-mediated demethylation of methylmercury can also occur via selenium amino acids, but this process has only been verified in a laboratory setting and has not been demonstrated in natural aquatic environments [72,73].

Atmospheric dimethylmercury is photodegraded to mercury and hydrocarbons [74]. Phenylmercury and sulphur-bonded methylmercury compounds (e.g., CH_3_HgS^−^) also decay photolytically quite quickly.

The photodemethylation function is only comparatively remarkable for surface waters because of the fast decay produced by light in the aqueous body, particularly by UV radiations [75,76,77]. The speed and efficiency of photodemethylation depend greatly upon the intensity and wavelength of the radiation; Short-wave UV-B light (280–320 nm) degrades methylmercury far more efficaciously than long-wave UV-A light (320–400 nm) and either visual light or photosynthetically active radiation (PAR) (400–700 nm). In a study on photodemethylation in taiga lake wetlands, the relative ratio of the demethylation rate constant to UV-B, UV-A, and PAR was found to be 3100:43:1 in surface water [78]. Similar results were found in a study by Black et al., where the photodemethylation rate constant for UV-B in water was at least 400 times that of PAR and 37 times that of PAR for UV-A [79]. In a research program on the effects of natural solar spectra and UV radiation on the range and characteristics of mercury isotopes, UV-B radiation was found to be the main factor in photodemethylation, with UV-A contributing little to the photodemethylation process [80]. The observed close relationship between Hg isotopes and incident radiant energy suggests that the Hg isotope signal can potentially serve as a useful instrument for quantifying the Hg photochemical cycles. Mercury isotopes have been used to trace the origin of MeHg and its biodegradation in marine organisms [81], and approximately 56–80% of MeHg can be photodegraded before it enters the food chain [82,83,84]. The various pathways of demethylation and their mechanisms and characteristics are shown in Table 2.

## 4. Factors Influencing Mercury Methylation and Demethylation

### 4.1. Redox Conditions

An environment in which the ambient oxygen level is lower than normal is known as a hypoxic environment. After decades of research, hypoxic environments have been identified as a major environment for methylation. Since most sediments are anoxic, contemporary studies continue observing significant anoxic in-sediment conditions for their methylation potency [89]. A reduction reaction is one in which a substance (molecule, atom, or ion) gains electrons or electron pairs in close proximity, while an oxidation reaction is the opposite. However, an increasing number of studies have shown that methylation could occur in both reducing and oxidizing environments. For example, methylation of mercury may occur in oxidizing marine surficial waters and polar marine waters [90,91,92]. Liu et al. found no relationship between MeHg levels and hypoxic tendencies in Gulf of Mexico water, despite higher underlying methylation and demethylation rates than in other littoral regimes [93]. Methylation of the roots of large plants in lakes in the tropics has only been seen in lakes under oxidizing conditions [94]. Oxidation conditions might even affect methylation in sediments, as higher levels of methylation have been observed in the higher layers of sediments below the oxygenated water layer, and conversely, lower methylation has been observed in sediments below the anoxic water layer [95,96]. A likely interpretation linking oxidative conditions to methylation may be that several methylating bacteria are not anaerobes and that they require oxygen [97]. In summary, methylation can occur under both anoxic and oxidative conditions.

### 4.2. Organic Substances

Organic matter can promote methylation by stimulating microbial activity. Different studies have shown that organic matter generated in wetland environments could chelate with methylmercury, thus enabling it to migrate to surface waters. It has been shown that an increase in dissolved organic carbon (DOC) concentration leads to a decrease in the specific rate of net methylation, which may be due to the complexation of inorganic mercury with DOC, and the effect is the greatest when the pH is reduced from 7.0 to 5.0 [98]. However, it could also chelate with inorganic mercury, thus reducing the bioavailability of mercury [99]. Several recent studies have demonstrated a clear correlation between some of the organic substances and methylmercury concentrations. For example, very high concentrations of methylmercury in water have been found in highly humified environments in New York’s freshwater marshlands in the Adirondacks [100]. Additionally, organic matter also has the possibility to promote the release of mercury from insoluble mercury sulphide, especially dissolved organic matter of the aromatic group [101]. All in all, organic substances act in multiple ways, by stimulating microbial activity, providing methyl groups for methylation, and via their ability to release mercury from cinnabar to promote and enhance the methylation of mercury, thus potentially methylating it.

### 4.3. Sulphide

In multiple water settings, different studies have found a strong relationship between the presence of sulphide and the levels of methylmercury. Since sulphate-reducing bacteria (SRB) are partially restricted in their activity by the presence of sulphate, an increase in sulphate could stimulate methylation [102,103]. Low concentrations of sulphide form neutrophilic mercuric sulphide compounds that diffuse via cell membranes, thus facilitating methylation, while higher levels of sulphide promote the formation of mercuric sulphide compounds and decrease the mercury’s bioavailability [104]. In a recent experiment, by manipulating the atmospheric sulphate loading of small peatlands, it was demonstrated that both methylmercury concentration and methylmercury percentage in pore water increased within one week after the addition of sulphate and decreased with the disappearance of sulphate. If the addition of sulphate was discontinued, the irritating effect on methylmercury was reduced. However, it is worth mentioning that, even in the past four years, the concentration and percentage of methylmercury were still higher than those in the control system [105,106]. Furthermore, Mitchell et al. investigated how both SO and organic C control net MeHg production using a controlled factorial addition design in 44 in situ peatland mesocosms and discovered that, although the addition of consumable OC to peat did not stimulate methylation, sulphate, either alone or in conjunction with some forms of OC, significantly enhanced methylation production in a related experiment [107]. Although there is still a clear correlation between sulphate presence and the potential for methylation, ongoing research suggests that methylation is directly correlated with the presence of sulphide. However, mercury circulation and methylation in the natural high-sulphate zone have not been fully surveyed, and studying the mechanisms of mercury transport, transformation, and accumulation in high-sulphate environments could be a potential area for future studies.

### 4.4. Temperature

Water temperature affects mercury biomethylation, as a positive correlation was found between the rate of methylmercury production and temperature [108,109,110,111]. Due to the absence of heat cycling, methylation occurs in the hypolimnion of lakes only under prolonged low temperatures [90]. However, the connection of methylation to low temperature is not clear. Methylmercury concentrations and methylmercury ratios are lower in subarctic lakes compared to lakes in the code area, possibly because of the lower temperatures, which are unsuitable for methylation, but also because of the different patterns of mercury deposition at different sites [112]. Therefore, the effect of temperature upon methylation is likely to depend upon the climate conditions of the particular water environment under study. Besides this, complex variables like enhanced hypoxia in deeper aquifers make it very hard to fully evaluate the effect of temperature on methylation.

### 4.5. pH

There is also much interest in the impact of pH on Hg methylation, particularly of the acidification of lake water due to atmospheric deposition. Numerous researchers have noticed enhanced mercury levels among species of fish in acidified lakes and are concerned that exposure to low pH might result in enhanced production and bioaccumulation of methylmercury. Modelling results suggest that the detected negative correlation of lake pH with fish Hg levels is because of a combination of prevailing higher methylmercury levels at low pH and lower bio-enrichment factors at high pH [113]. However, changes in pH could affect methylmercury concentrations in aquatic systems in many ways, whereas the direct influence of pH on the rate of methylation is uncertain. For example, the dissolution and fluidity of mercury and methylmercury depend on the pH value, but acidic rain or snow might increase the mercury input of the watershed [114].

### 4.6. Iron and Manganese Oxides

Recent studies have demonstrated that both Fe(II) and Fe(III) oxides, as well as manganese oxides, exert significant influence on the cycling and methylation of mercury through different mechanisms. Notably, elevated methylation levels were detected in areas rich in dissolved Fe^2+^ and organic materials within wastewater treatment facilities, where water undergoes purification via hydrated iron oxide [115]. This observation suggests that Fe(II) may directly facilitate enhanced mercury methylation.

Conversely, Fe(III) oxides seem to impact mercury cycling and methylation through indirect pathways. Research indicates that compounds loaded with hydroxyl radicals, produced by Fe(III) oxides via Fenton-like reactions, can serve as methyl donors during photomethylation processes. While these compounds can augment methylation at low concentrations, excessive amounts may lead to the degradation of methylmercury [116].

Investigations across various lake systems reveal that mercury transformation is influenced by the redox cycling of other elements, independent of the mercury source. This influence is particularly pronounced for redox-sensitive and microbiologically important elements such as sulphur, iron (both Fe(II) and Fe(III)), and manganese, along with their interactions with organic matter [117]. The connection between element availability and methylation in certain aquatic environments may relate to the role of these elements in facilitating microbial activity, which in turn affects methylation trends.

The biological methylation process of mercury is intricately linked to the dissimilatory reduction of Fe^3+^ to Fe^2+^, with iron-reducing bacteria (DIRB) playing a crucial role as mercury-methylating microorganisms. The microbial methylation efficiency of mercury typically correlates with microbial activity and is further modulated by factors such as temperature, pH, redox conditions, sulphide concentrations, and various organic chelating agents. Table 3 illustrates the six principal factors influencing methylation in the water column, along with their specific effects.

## 5. Conclusions and Outlook

Methylation and demethylation of mercury are important components of the methylmercury cycle and determine methylmercury levels in aquatic ecosystems. They can arise in different parts of aquatic ecosystems, in sediment, in epiphytes, and in the water. Biogenic methylation in sediments is the main source of methylmercury in the water environment.

The biological methylation of mercury is either enzyme-promoted or non-enzyme-promoted, and the process of microbial methylation of mercury requires the involvement of methylcobalamin. The likelihood of microbial methylation of Hg is usually higher under anaerobic conditions, and three species of bacteria, methanogenic, iron-reducing, and sulphate-reducing bacteria, are thought of as the major bacteria species involved in this process, with the hgcA and hgcB genes playing an essential role in the process of Hg methylation by some anaerobic bacteria. Mercury can also be purely chemically methylated, like in photochemical methylation, if a suitable methyl donor is available. Demethylation of methylmercury can occur via both biotic and abiotic pathways. Mercury-resistant bacteria with genes for mer-AB manipulators can dominate the demethylation process. Sulphate-reducing bacteria (SRB) and methanogens may be the main microorganisms involved in anaerobic demethylation, and a new pathway for biodegradation of methylmercury exists in methanogens that is distinctly different from the typical organic Hg lytic enzymes found in some aerobic microorganisms. In addition, UV-B light degrades methylmercury more efficiently than UV-A light and visible light, and photolysis is also an important route for methylmercury demethylation. Methylation and demethylation procedures are affected by a variety of elements. Methylation usually occurs under anoxic conditions, but in some cases, it can also take place in an oxidative environment. Organic matter promotes methylation and correlates with methylmercury concentrations. The presence of sulphide is strongly correlated with methylmercury levels, with low concentrations of sulphide promoting methylation and high concentrations reducing mercury bioavailability. Temperature has an effect on the rate of methylation, with methylmercury being produced more rapidly at higher temperatures. Low pH may result in increased methylmercury production and bioaccumulation. Iron and manganese oxides have a significant effect on methylation, with compounds containing hydroxyl radicals enhancing methylation. These factors interact and together determine the processes of methylation and demethylation. Their effects may vary under different aquatic environments and climatic conditions, depending on which factors dominate. Methylation by methanogenic bacteria seems to have been neglected due to the discovery of the mercury methylation capacity of sulphate-reducing bacteria, and this has led to an underestimation of the important role of methanogenic bacteria in the methylation of mercury. It is, therefore, important and forward-looking to strengthen research on the role of methanogenic bacteria in the methylation of mercury. The discovery of hgcAB gene pairs has made the principle of mercury methylation of microorganisms clearer, but the relative rarity of microorganisms possessing hgcAB gene pairs further increases the difficulty of detection and quantification. Future research on the protein structure, molecular mechanism, and biological significance of the hgcAB gene pair should be conducted.

## Figures and Tables

**Table 1:** Methylmercury levels in some objects.

Type	Average Methylmercury Content	Reference
Freshwater Crab	0.028 mg/kg	[32]
Freshwater Fish	0.034 mg/kg	[32]
Marine Fish	0.031 mg/kg	[32]
Rice	0.00137 ± 0.00118 mg/kg	[33]
*Beryx splendens*	0.78 ± 0.56 mg/kg	[34]
Atlantic *Thunnus thynnus*	0.42 ± 0.06 mg/kg	[34]
*Thunnus obesus*	0.98 ± 0.34 mg/kg	[34]
*Tetraptrus audax*	0.51 ± 0.08 mg/kg	[34]
*Hypophthalmichthys moritrix*	0.18 ± 0.09 mg/kg	[35]
Sediment	0.06 mg/kg to 1.38 mg/kg	[35]
Australian Reed	0.618 mg/kg	[36]
Carps	0.019 mg/kg to 0.063 mg/kg	[37]
Long-tailed Tuna	0.180 mg/kg to 1.460 mg/kg	[38]
Sri Lankan Rice	0.0051 ± 0.37 mg/kg	[39]

**Table 2:** Comparison of demethylation pathways for mercury in the aquatic environment.

Type of Demethylation	Mechanism of Action	Characteristics	References
Mer operon demethylation	Genes encode lytic and reductive enzymes that lyse methylmercury to methyl and mercury(II) and reduce mercury(II) to mercury(0)	Widespread in mercury-resistant bacteria	[61,62]
Demethylation of methane nutrients	Bacteria produce methanobactin molecules to promote methane oxidation and methylmercury degradation	Uses the methyl group in methylmercury as an auxiliary C1 carbon source for microorganisms	[65]
Phytoplankton demethylation	Phytoplankton utilise endogenous reactive oxygen species as the main driver of demethylation	Can degrade methylmercury by dark reaction	[66,67]
Selenium amino acid demethylation	Methylmercury and selenoamino acids through the formation of bis(methylmercury) selenide and dimethylmercury as intermediates, with HgSe(s) as the final degradation product	Laboratory stage, not proven in natural environment	[70,71]
Photodemethylation pathway 1	Methylmercury causes C-Hg bond breaking by direct absorption of light energy	Most important demethylation pathway in the aquatic environment, about 56–80% of methylmercury is photodegradable (pathway1, 2, 3)	[85]
Photodemethylation pathway 2	Reactive oxygen species (ROS) and other photoactive substances, produced by organic molecules, ions, suspended solids, etc., attack the C-Hg bond and degrade MeHg when exposed to sunlight		[86,87]
Photodemethylation pathway 3	When MeHg is complexed with photochemically active dissolved organic matter (DOM) and exposed to light, the excited DOM-MeHg complex may undergo intramolecular electron transfer, leading to C-Hg bond breaking		[88]

**Table 3:** Factors influencing methylation in the water column and their effects.

Influencing Factors	Effects on Methylation
Redox conditions	The anoxic environment is the primary environment for methylation, but methylation under anoxic and oxidative conditions both may occur.
Organic substances	Several recent studies have demonstrated a clear correlation between certain organic substances and methylmercury concentrations. Organic substances act in a number of ways, by stimulating microbial activity and by providing methyl groups.
Sulphide	Increased sulphate may stimulate methylation.
Temperature	The rate of methylmercury production is generally positively correlated with temperature.
PH	May be negatively correlated, with higher levels of methylmercury prevalent at low pH.
Iron and manganese oxides	Methylation levels are highest in areas rich in dissolved iron and organic matter, but it is still not possible to determine their exact effect or impact.

## Data Availability

The study did not report any data.
